# Evolution of the C-Type Lectin-Like Receptor Genes of the DECTIN-1 Cluster in the NK Gene Complex

**DOI:** 10.1100/2012/931386

**Published:** 2012-04-01

**Authors:** Susanne Sattler, Hormas Ghadially, Erhard Hofer

**Affiliations:** ^1^Department of Vascular Biology and Thrombosis Research, Medical University of Vienna, 1090 Vienna, Austria; ^2^Division of Immunology and Infection, Department of Medicine, CCIR, Imperial College London, London W12 0NN, UK

## Abstract

Pattern recognition receptors are crucial in initiating and shaping innate and adaptive immune responses and often belong to families of structurally and evolutionarily related proteins. The human C-type lectin-like receptors encoded in the DECTIN-1 cluster within the NK gene complex contain prominent receptors with pattern recognition function, such as DECTIN-1 and LOX-1. All members of this cluster share significant homology and are considered to have arisen from subsequent gene duplications. Recent developments in sequencing and the availability of comprehensive sequence data comprising many species showed that the receptors of the DECTIN-1 cluster are not only homologous to each other but also highly conserved between species. Even in *Caenorhabditis elegans*, genes displaying homology to the mammalian C-type lectin-like receptors have been detected. In this paper, we conduct a comprehensive phylogenetic survey and give an up-to-date overview of the currently available data on the evolutionary emergence of the DECTIN-1 cluster genes.

## 1. C-Type Lectin-Like Receptors

Receptors on the surface of immune cells, which bind to molecular patterns common to certain classes of pathogens, such as components of bacterial or fungal cell walls, play a crucial part in the immediate innate immune response to these pathogens as well as in shaping and regulating the subsequent adaptive immune response. Innate immune signaling via pattern recognition receptors, such as the Toll-like receptors [1], has even received media attention recently, as the 2011 Nobel prize for medicine was awarded to Bruce A. Beutler and Jules A. Hoffman “for their discoveries concerning the activation of innate immunity” [2, 3].

Similar to the Toll-like receptors, other pattern recognition receptors such as certain C-type lectin-like receptors are structurally and evolutionarily related members of protein families, which have arisen from gene duplication and expansion due to the high evolutionary pressure applied by pathogens [4–6]. C-type lectin receptors were originally defined by their carbohydrate-binding function, which enables them to bind to complex oligosaccharides displayed on various biological structures such as cell surfaces, circulating proteins, and extracellular matrices. By binding to these structures, C-type lectins mediate a variety of crucial cellular processes including cell adhesion, serum glycoprotein turnover and quick innate-type immune responses to potential pathogens [7]. In contrast to other animal lectins, carbohydrate binding by these proteins was initially found to be dependent on calcium ions; therefore this receptor family was termed “C-type” lectins. Later it became evident that not all family members bind to carbohydrates exclusively and that, for some, binding might also be independent of calcium, so the designation of C-type lectin-like receptors was introduced [8].

One well-studied family of C-type lectin-like receptors is encoded in the natural killer gene complex (NKC) on the short arm of human chromosome 12 [9]. The most prominent receptor family in this genomic region is the NK cell group 2 (NKG2) family, which owes its name to a preferential expression on NK cells [10]. This gene cluster has been analyzed thoroughly by our group and others [11–14], and data available on its evolutionary development has been reviewed previously in great detail [15].

However, at the telomeric side of the NKG2 gene cluster a subfamily of genes expressed in different myeloid as well as nonimmune cell types rather than on NK cells has been identified [16, 17]. This cluster has originally been termed myeloid cluster of the NKC [16, 17] and is now also referred to as DECTIN-1 cluster. It comprises genes encoding well-studied proteins like DC-associated C-type lectin-1 (DECTIN-1), lectin-like oxidized low-density lipoprotein receptor-1 (LOX-1), C-type lectin-like receptor-1 (CLEC-1), and CLEC-2 [18]. Recently, we and others have identified and characterized the additional C-type lectin-like receptors CLEC9A and CLEC12B, which belong to the DECTIN-1 subfamily of this region [19–21].

## 2. Evolution of the C-Type Lectin-Like Receptors of the NK Gene Complex

The C-type lectin (CLECT) motif for carbohydrate recognition has emerged early during evolution. It can be found in proteins of many model organisms, including the yeast *Saccharomyces cerevisiae*, the nematode *Caenorhabditis elegans*, and the fruit fly *Drosophila melanogaster *[22]. However, homology between these proteins and the mammalian C-type lectin-like receptors of the NK gene complex are restricted to the CLECT domain (CTLD).

**Figure 1:**
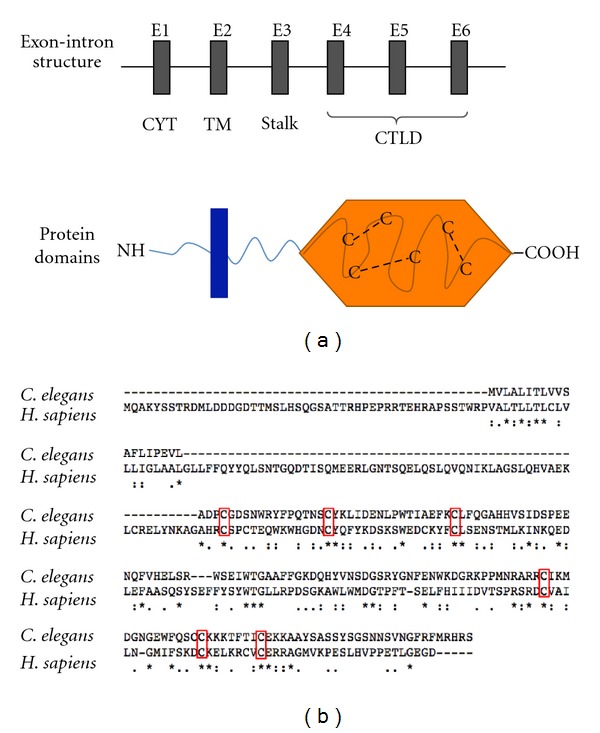
Evolutionary conserved characteristics of C-type lectin-like receptors. (a) Schematic representation of the common exon-intron structure of C-type lectin-like receptors and the corresponding protein domains. N-terminal cytoplasmic domain, transmembrane, and stalk region are each encoded by one exon, the C-type lectin-like domain (CTLD) by three exons. Disulfide bridged bonds between six highly conserved cysteins in the extracellular CTLD cause the typical *β*-sheet tertiary structure of C-type lectin-like proteins. (b) Alignment of human and putative *C. elegans* CLEC-1 protein sequences. Conserved CTLD cysteins are boxed in red, asterisk (*) fully conserved, colon (:) strongly similar properties (>0.5 Gonnet PAM 250 matrix), period (.) weakly similar properties (>0.5 Gonnet PAM 250 matrix). Alignments were produced using ClustalW [26]. CYT: cytoplasmic region, TM: transmembrane region, CTLD: C-type lectin-like domain.

As shown in Figure 1, mammalian C-type lectin-like receptors share a very specific and highly conserved common protein structure. They are type II transmembrane proteins and consist of a short N-terminal cytoplasmic domain, a transmembrane region, a stalk domain providing a flexible linker between transmembrane and ligand-binding domain, each encoded by a single exon (E1–E3), and the C-terminal extracellular ligand-binding CTLD encoded by three exons E4-E5. The most highly conserved feature of C-type lectin-like receptor proteins consists of six cysteine residues which are located in the external ligand binding domain. This high conservation, an example of which is shown in Figure 1(b) by an alignment of the human and the putative *C. elegans* CLEC-1 protein, is accordant with the fact that these cysteine residues are involved in intrachain disulfide bonds forming the characteristic C-type lectin-like fold [23, 24], which is crucial for ligand binding, internalization and phagocytosis as demonstrated for LOX-1 [25]. However, it is likely that the *C. elegans* protein, although termed CLEC-1, only displays the common characteristics of C-type lectin-like receptors and therefore might represent an ancestral progenitor of the DECTIN-1 cluster genes, rather than being an ortholog of one specific receptor.

**Figure 2:**
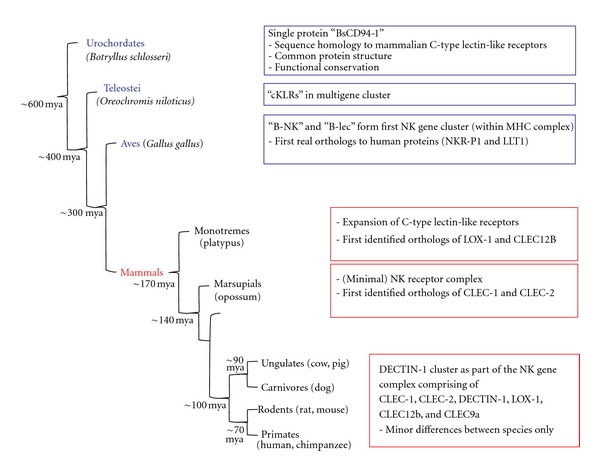
Evolutionary emergence of the C-type lectin-like receptors of the DECTIN-1 cluster. Schematic presentation of the evolutionary emergence of the members of the DECTIN-1 cluster. First transmembrane C-type lectin-like receptors appear in evolutionary old species like urochordates. However, orthologs of receptors of the DECTIN-1 cluster are restricted to mammals. Numbers indicate approximate divergence times in million years. cKLR: cichlid killer cell C-type lectin-like receptors, NKR-P1: natural killer receptor-P1, LLT1: lectin-like transcript-1, MHC: major histocompatibility, mya: million years ago.

Figure 2 depicts a summary of the currently available data on the evolutionary relationship between C-type lectin-like receptors. The evolutionary oldest species which has been shown to possess a C-type lectin-like protein with the above-mentioned conserved sequence characteristics is the urochordate (sea squirt) *Botryllus schlosseri,* a colonial invertebrate chordate. The protein BsCD94-1 is a type II transmembrane receptor with a C-type lectin-like domain with highest similarity to the mammalian C-type lectin-like receptors natural killer receptor-p1 (NKR-P1), NKG2D, and CD94. Similarity to the mammalian receptors was even higher than to previously identified soluble urochordate lectins. The protein was named BsCD94-1, because its short intracellular region resembles the 7-amino acid cytoplasmic domain of mammalian CD94. Interestingly, BsCD94-1 is expressed on a subset of *B. schlosseri* blood cells and has a role in allorecognition. This indicates not only that the receptor is also conserved on a functional level, but also that these urochordate blood cells might belong to an ancestral cell population which represents the evolutionary origin of NK cells [27].

The bony (cichlid) fish *Oreochromis niloticus* contains C-type lectin-like genes, termed cichlid killer cell C-type lectin-like receptors (cKLRs), that are already arranged in a multigene cluster resembling the mammalian NK gene complex and harbor a transmembrane region, a stalk domain and a CTLD. Phylogenetic and protein domain analysis reveals a close phylogenetic relationship of the cKLRs to the receptors of the mammalian NK gene complex. However, as for BsCD94-1 in *Botryllus schlosseri,* no orthology to one particular receptor is apparent [28]. Therefore, the first C-type lectin-like receptor genes with expression on a putative NK cell precursor population seem to have arisen before the divergence of fish and tetrapods >400 million years ago with subsequent independent duplications of a common ancestral gene [28]. 

The first nonmammalian proteins with clear homology to one specific mammalian receptor encoded in the NK gene complex have been described in chicken (*Gallus gallus*). Six C-type lectin-like NK receptor genes have been identified within two distinct regions of the chicken genome. One of these regions shares conserved synteny with the human NK cell receptor complex suggesting that not only single CTLD containing genes but the whole chromosomal region existed already before the divergence of mammals and birds ~300 million years ago [29–32]. Two chicken C-type lectin-like receptors, B-NK and B-lec, have been characterized and show the common C-type lectin-like exon-intron as well as protein domain structure. B-NK and B-lec are most closely related to the mammalian NKR-P1 and lectin like transcript-1 (LLT1), respectively. Interestingly, in contrast to the NK gene complex in mammals, which forms an entirely independent entity from the major histocompatibility (MHC) complex, B-NK and B-lec which comprise the whole chicken NK gene complex are encoded within the MHC cluster, suggesting that the MHC complex and NKC share a common evolutionary origin [32].

Among evolutionary older mammals, marsupial mammals (opossum, *Monodelphis domestica*) [33] and monotremes (platypus, *Ornithorhynchus anatinus*) [34] have been shown to possess an NK gene complex. Monotremes diverged from placental mammals 167 to 218 million years ago [35, 36], marsupials 148 to 190 million years ago [36, 37]. The marsupial NK gene complex is less complex as compared to evolutionary younger mammals. However, there are obvious orthologs to genes existing in the primate NK gene receptor complex, such as CD69, CLEC-1B, CLEC-1A and killer cell lectin-like receptor subfamily K, member 1 (KLRK1) coding for the NKG2D receptor [33]. In platypus on the other hand the NK gene complex has expanded and split into at least two regions of the genome. 213 putative C-type lectin-like receptor genes have been identified, which may have arisen from lineage-specific expansion. Among these genes, orthologs of LOX-1, CD69, killer cell lectin-like receptor-1 (KLRE-1), and CLEC12B have been identified [34]. Evolutionary younger mammals, like ungulates, rodents and nonhuman primates, share many orthologous C-type lectin-like receptors, and syntenic regions encoding the NK gene complex have been identified in most genomes [38].

## 3. The C-Type Lectin-Like Receptors of the DECTIN-1 Cluster

**Figure 3:**
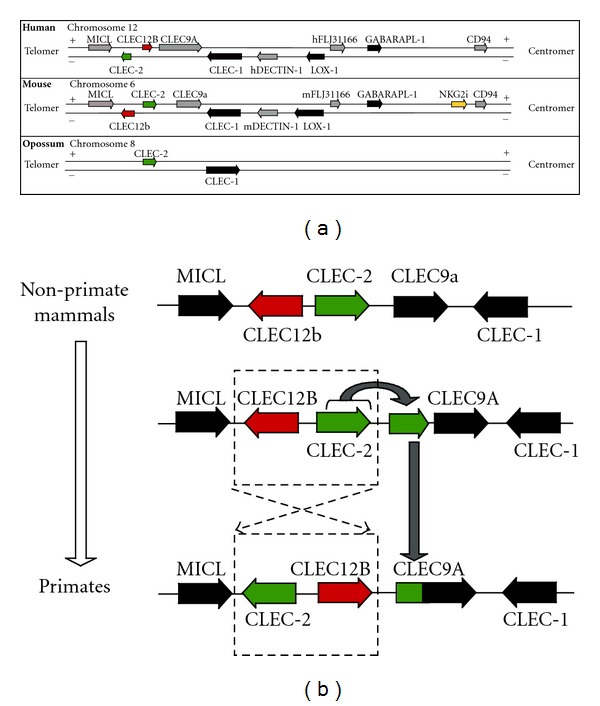
Organization and transcriptional orientation of the genes in the human, mouse and opossum DECTIN-1 cluster. (a) Physical map comparing the DECTIN-1 cluster in the centromeric part of the human NK complex with the corresponding region of the mouse and opossum NK complex, extending from the myeloid inhibitory C-type lectin-like receptor (MICL) gene to the CD94 gene. Linkage and orientation of the genes in the myeloid cluster of the NK gene complex are depicted. (b) Possible course of events leading from the non-primate to the primate mammalian configuration of the myeloid cluster between the genes encoding MICL and CLEC-1. Modified and reprinted with permission of John Wiley & Sons, Inc. © 2010 from [21].

The myeloid or DECTIN-1 cluster in the centromeric part of the NK gene complex (Figure 3(a)) stands apart from the cluster of NK receptor genes in this region. Although homologous in sequences, the receptors of the DECTIN-1 cluster differ from the receptors of the NKG2A and Ly49 family in terms of their protein expression pattern and function in the immune system. NKG2 family members are predominantly expressed on NK cells and a fraction of T lymphocytes. They recognize nonclassical MHC class I and related molecules on target cells and thereby monitor appropriate self-MHC expression [39]. Members of the DECTIN-1 cluster on the other hand are usually expressed on various myeloid cells and some also on endothelial cells, but not on NK cells or T lymphocytes [16]. They have pattern recognition function as well as various other immune as well as nonimmune functions. Furthermore, members of the DECTIN-1 family are evolutionarily closer related to each other than to members of the NK subfamily and the encoding genes are physically separated by a longer stretch of noncoding genomic DNA [21].

As mentioned above, the first orthologs of DECTIN-1 cluster genes appear together with members of the NKG2 family in marsupial mammals (CLEC-1B, CLEC-1A) and monotremes (LOX-1, CLEC12B) [33, 34].  Table 1 summarizes in which species DECTIN-1 cluster members have been identified and how much information is available about the respective receptors.  Table 2 summarizes available information on ligands and functions as well as conservation between the receptors.

As we have shown previously [21], the DECTIN-1 cluster is a genomic region with a high evolutionary activity in more recent times indicated by a high AluS : AluJ ratio of 5,25 : 1 compared to a whole genome ratio of 3 : 1 [5] as well as a high number of evolutionary young AluY elements. Alu sequences are abundant mobile elements propagating through retrotransposition, and the ratio between numbers of Alu subfamily members (AluS, AluJ, and AluY) can be used as a measure of the evolutionary age of a specific genomic region [40]. AluY elements were active about 24 million years ago and are therefore the most recently active Alu repeats [41]. The AluS subfamily was active about 30 million years ago after the divergence of the primate from the rodent lineage, whereas AluJ repeats diverged 60 million years ago from a common source element [42]. Therefore, the ratio between Alu elements of different subfamilies indicates that the DECTIN-1 cluster showed relatively recent evolutionary activity. Despite the recent activity of Alu sequences, the order and orientation of most genes in the DECTIN-1 cluster seem to be preserved between human and mouse except in a region of about 40 kb containing the CLEC-2 and CLEC12B genes (Figure 3(b)). This region seems to be inverted in genomic sequences of primate as compared to nonprimate mammals. Additionally, sequences highly homologous to CLEC-2 are present in the genomic region directly upstream of the human CLEC9A gene. This suggests a duplication of the CLEC-2 gene followed by an inversion of the genomic region containing the complete CLEC-2 and CLEC12B genes in a common primate ancestor, which is in line again with recent evolutionary changes in this area.

Whereas the C-type lectin protein motif can be found in organisms of most evolutionary branches, C-type lectin-like receptors as defined by Weis et al. [43] seem to appear only in members of the chordate phylum, including mammals, birds, and fish. However, known orthologs to receptors of the DECTIN-1 cluster are limited to mammals.

## 4. DECTIN-1

DECTIN-1 has first been described in mouse [44], but soon after that homologs have been identified in human [16, 45], as well as in cow [46] and pig [47]. As expected, other primate as well as other rodent species express DECTIN-1 as well. Full-length human DECTIN-1 (isoform A) shares 72% DNA [48] and 61% protein sequence identity with the murine receptor. The homology to the bovine protein sequence is 74%, to the predicted chimpanzee DECTIN-1 sequence 98%, and to the canine sequence 74% (Table 2). Since its initial discovery, DECTIN-1 has been thoroughly characterized and is now well known to be a major receptor for *β*-glucan, a common component of fungal cell walls. It is expressed on myeloid cells of the innate immune system and thereby plays a key role in innate immune defense against fungal pathogens [49]. Upon ligand binding, intracellular signaling via a cytoplasmic immunoreceptor tyrosine-based-activation-motif (ITAM)-like motif recruits Syk kinase resulting in activation of nuclear factor-*κ*B (NF-*κ*B), mitogen-activated protein kinase (MAPK) and nuclear factor of activated T cells (NFAT) [50, 51].

In most species, DECTIN-1 mRNA appears in form of two major splice variants, termed isoform A and B, which are the only isoforms that can bind *β*-glucans [46, 47, 49]. These splice variants are translated into proteins with or without a stalk domain, respectively, and it has been suggested that stalkless isoform B is less efficient in ligand binding and that the use of differential isoforms might be a mechanism of regulating cellular responses to *β*-glucan [52]. In addition, at least 8 more variations of the A and B isoforms generated by alternative splicing have been described but are predicted to produce truncated proteins [49, 53]. Furthermore, isoform E, which has only been identified in humans, lacks the transmembrane region and therefore is retained in the cytoplasm instead of being transported to the cell surface [54].

Interestingly, DECTIN-1 has been shown to have clinical relevance in the context of fungal infections. DECTIN-1-deficient mice showed increased susceptibility to oral candidiasis, with enhanced dissemination and decreased survival times [55]. In humans, a polymorphism of human DECTIN-1 (Y238X) with an allele frequency of 3–7% in populations from Africa and western Eurasia has been identified. The polymorphism causes an early-stop-codon mutation in the DECTIN-1 CTLD, resulting in defective protein expression and lack of *β*-glucan recognition by phagocytes leading to impaired cytokine production. Clinically, this mutation results in recurrent fungal infections in the carriers [56]. It has also been reported that the Y238X DECTIN-1 polymorphism is associated with increased susceptibility to oral and gastrointestinal colonization with *Candida* species in hematopoetic stem cell transplant (HSCT) recipients [57]. Possible polymorphisms have also been identified in other species, such as sheep, and it has been speculated that these polymorphisms might be implicated in increased susceptibility to fungal infections [58]. Therefore, DECTIN-1 might not only be a relevant factor in human healthcare, but might also have important implications for maintenance of domestic and live stock animals.

## 5. LOX-1

LOX-1 was first identified in both bovine (protein sequence homology to human 73%) and humans [59] quickly followed by mouse (61%) [60]. LOX-1 homologs are now also known to exist in pigs [61], rats [62], and rabbits [63]. Predicted sequences derived from automated computational analysis of sequences with high homology to the human and rodent proteins are also available for canine LOX-1 (74% protein identity to human LOX-1). Human and the corresponding predicted chimpanzee LOX-1 sequences share 99.3% protein identity. Strikingly, an ortholog of human LOX-1 with a protein identity of 35% has recently been identified in platypus (Table 2). The protein has a predicted CTLD which contains six cystein residues [34]. However, SMART protein domain analysis fails to detect a transmembrane region in the currently available sequences [64].

LOX-1 has been shown to bind a variety of negatively charged ligands, the most prominent being modified lipoproteins [60], aged/apoptotic cells [65] and Gram-positive and Gram-negative bacteria [66]. Interestingly, LOX-1 lacks classical C-type lectin-like signaling motifs, such as tyrosine residues or transmembrane cationic amino acids [67]. Nevertheless, activation of LOX-1 upon binding to one of its ligands has been implicated in various cellular functions, including endocytosis, phagocytosis, apoptosis, activation of the NF-*κ*B pathway, and production of cytokines and reactive oxygen species (ROS) [68–70]. An isoform of LOX-1 generated by alternative splicing has been termed LOXIN and was described to completely lack a ligand-binding domain [71]. Similar to stalkless DECTIN-1, LOXIN has been demonstrated to downmodulate LOX-1 activity by hetero-oligomerization with the active full-length isoform. Full-length LOX-1 protein can be cleaved by a serine protease between Arg86-Ser87 and Lys89-Ser90 in its membrane proximal extracellular domain and thereby sheds a soluble form of the protein [72, 73]. To date, only CLEC-2 has beed demonstrated to be shed by a similar mechanism [74]. LOX-1 biology has been extensively studied because of its role in vascular diseases like atherosclerosis, thrombosis, myocardial ischemia reperfusion injury, hypertension as well as its involvement in generating inflammatory responses during microbial infections [69, 75]. Especially circulating levels of soluble LOX-1 (sLOX-1) in human serum have been found to be increased in inflammatory and atherosclerotic conditions. They are associated with acute coronary syndrome, the severity of coronary artery disease and with the serum levels of biomarkers for oxidative stress and inflammation [76, 77]. These correlations lead to the suggestion that LOX-1 could be used as a novel biomarker to predict vascular injury [78]. Although measurement of soluble LOX-1 may provide a useful diagnostic tool, the endogenous function of sLOX-1 is still unclear, as preliminary data suggest that sLOX-1 does not seem to act as an inhibitory decoy receptor [72].

Clinically relevant polymorphisms of LOX-1 have also been identified. A gene polymorphism (G501C) resulting in a missense mutation (K167N) in the LOX-1 protein has been associated with myocardial infarction [79] and coronary artery disease [80]. The underlying mechanism was determined to be a decrease in ligand binding affinity, leading to reduced oxidised low-density lipoprotein (ox-LDL) binding and uptake [81]. LOX-1 polymorphisms have not been reported in other mammals so far. However, it is likely that similar to DECTIN-1, other species also have polymorphisms in LOX-1. Whether these polymorphisms turn out to have any relevance in the context of live stock or domestic animal health remains to be investigated.

## 6. CLEC-1

Expression of CLEC-1 has first been described in humans [16, 82] and later in rodents [21, 83]. Although poorly characterized, CLEC-1 appears to be an important protein judging from its high evolutionary conservation of 70 percent between the human and mouse protein [84]. Opossum possesses a minimal NK gene complex with only nine C-type lectin-like receptor genes in total, and only CLEC-1 and CLEC-2 as members of the DECTIN-1 cluster [33, 85]. However, the available sequences contain only the gene region encoding the CTLD of these receptors. Alignment of these sequences to the human sequence results in a similarity of 74%. Predicted *Bos taurus* and *Canis familiaris* CLEC-1 both share 75% similarity with the human protein sequence, respectively (Table 2), and SMART protein domain analysis predicts proteins with a typical C-type lectin-like domain structure, including a CTLD, a coil-coil, and a transmembrane domain. However, experimental information on corresponding proteins is still missing. Human CLEC-1 and the predicted chimpanzee protein share 99.3% identity. Interestingly, mRNA sequences for a *Perca flavescence* (yellow perch) gene termed CLEC-1 have been generated from whole-genome and high-throughput cDNA sequencing [86, 87]. The sequence of the *Perca* CLEC-1 protein aligns to parts of both human and mouse CLEC-1 with a 17.1% score using the ClustalW algorithm [26]; however, the similarity to human DECTIN-1 is even slightly higher, with a score of 18.8%. A *C. elegans* protein also termed CLEC-1 has 12% similarity to the human CLEC-1 protein. SMART protein domain analysis predicts a CTLD but no transmembrane region in both proteins. Although the available sequences are probably only parts of corresponding full-length proteins, sequence comparisons indicate that *P. flavescence* and *C. elegans* CLEC-1 only share the common similarities between C-type lectin-like proteins because of the presence of the C-type lectin-like domain. They may not be real orthologs to human CLEC-1 but rather display the characteristics of an ancestral progenitor of the DECTIN-1 cluster genes.

CLEC-1 still is the least well-studied member of the DECTIN-1 family, as characterization and ligand identification proved difficult due to its cellular localization which seems to differ from other family members. In our hands, CLEC-1 could not be detected on the surface of different CLEC-1 expressing cells even after stimulation [88]. Also, in contrast to other family members, CLEC-1 may have an immune regulatory rather than a proinflammatory function, as it has been demonstrated that in rodents and humans CLEC-1 expression is upregulated in response to immunoregulatory cytokines [83, 88].

Human and rodent CLEC-1 contain one tyrosine residue in the cytoplasmic tail close to the N-terminus which could potentially be utilized for downstream signaling. Mouse and rat have an additional tyrosine residue forming the ITAM-like pattern YxxTx13YxxT [84]. However, how or whether phosphorylation of these tyrosines occurs and leads to downstream signaling has yet to be analyzed.

## 7. CLEC-2

CLEC-2 has been identified together with CLEC-1 in human myeloid cells [16, 82], and similar to CLEC-1, the only other species where CLEC-2 has been characterized so far is rodents [74]. Predicted sequences derived from automated computational analysis of sequences with high homology to the human and rodent proteins are available for canine and bovine CLEC-2 with 70% and 69% protein identity to the human receptor, respectively. Interestingly, CLEC-2 does not seem to be as highly conserved as CLEC-1 as even the conservation between human and the predicted chimpanzee CLEC-2 is only 96.9% as compared to 99.3% identity between human and chimpanzee CLEC-1. As mentioned before, opossums have a minimal NK gene complex containing putative orthologs of CLEC-1 and CLEC-2 [33, 85]. Available sequences contain only the region encoding the CTLD, and alignment of these to the human CLEC-2 sequence results in a similarity of 74% (Table 2).

CLEC-2 has been found to bind to the exogenous ligand rhodocytin, a snake venom, [89] and the endogenous ligand podoplanin, a protein expressed on lymphatic but not vascular endothelium [90]. Human CLEC-2 has one YXXL motif in its cytoplasmic tail which undergoes tyrosine phosphorylation by Src kinases upon ligand binding [89]. Binding of podoplanin by CLEC-2 expressed on platelets leads to platelet activation and has been implicated in tumor metastasis and lymphatic/blood vessel separation during development. Furthermore, it might also play a role in HIV transmission [91–93]. In mice, CLEC-2 has been shown to be expressed on neutrophils, where it mediates phagocytosis and proinflammatory cytokine production [94]. In this study, phagocytosis has been investigated using antibody-coated beads, however the pathogens recognized and taken up by CLEC-2, still have to be identified. Nevertheless, these results indicate that CLEC-2, like LOX-1 and DECTIN-1, could have innate immune pattern recognition function.

In contrast to humans which only express the full-length CLEC-2, mice seem to have two additional splice variants. mCLEC-2b lacks the transmembrane region, and further deletions in mCLEC-2c cause a frameshift resulting in a truncated protein. As these isoforms show different expression patterns and subcellular localization, they might serve different and potentially even regulatory functions [74]. No information about CLEC-2 polymorphisms and possible clinical relevance is available so far.

## 8. CLEC9A

CLEC9A is the most recently described member of the DECTIN-1 cluster, and has been identified in human dendritic cells (DCs) and monocytes [19, 21] as well as in mouse dendritic cells [95]. As for the other members of the family, sequences for homologous genes have been predicted to exist in dog (protein homology to human sequence: 72%), cattle (71%), and chimpanzee (98.8%) (Table 2).

We have shown previously that in all primate species analyzed, sequences highly homologous to CLEC-2 can be found in the genomic region directly upstream of the CLEC9A gene, and parts of the 5′-UTR sequence of the CLEC9A mRNA are clearly derived from a duplication of an ancestral CLEC-2 sequence. However, these sequences do not seem to contribute to the CLEC9A coding sequence [21].

Human CLEC9A is translated from 6 exons and the protein is expressed at the cell surface as a glycosylated dimer. In mice alternative splicing can generate five isoforms. One isoform shows the common structure of a six exon-derived C-type lectin-like protein and has been shown to be expressed on the cell surface as a nonglycosylated monomer [19]. In addition, a seven exon-derived isoform with an additional exon in the CTLD, is expressed on the surface as a homodimer [95]. Analogous to CLEC-2, it has been speculated that different isoforms differ in function and might even regulate expression or protein activities.

Ligands for CLEC9A have yet to be identified. However, signaling via CLEC9A involves its cytplasmic ITAM-like motif and recruitment of Syk, similar to DECTIN-1 signaling [19]. Recently, it has been shown that CLEC9A expressed on a subset of dendritic cells binds to an unidentified ligand on necrotic cells and mediates cross-presentation and T-cell responses to dead-cell-associated antigens [96].

## 9. CLEC12B

CLEC12B was identified in human macrophages [20], and correspondingly mRNA is expressed in monocytic cell lines and primary myeloid cells [21]. Sequences are available for CLEC12b in mouse (protein homology to human sequence: 72%) and predicted in dog (81%) and cow (80%). Interestingly, a putative ortholog of human CLEC12B has been identified in platypus [34], which shares 26% identity with human CLEC12B (Table 2). CLEC12B is unusual as it is the only known receptor within the DECTIN-1 cluster, which acts as an inhibitory receptor via recruitment of Src homology phosphatase (SHP)-1 and SHP-2 [20]. We have demonstrated that human CLEC12B has several splice variants, and many of them are likely to render the corresponding protein nonfunctional. However, as these isoforms are the variants with the highest mRNA expression levels in most cell types, it can also be speculated that they could still serve a function different from the full-length form [21].

## 10. Concluding Remarks

The C-type lectin protein motif can already be found in evolutionary very old species, whereas C-type lectin-like receptors containing the typical common protein structure appear later in members of the chordate phylum, with representatives identified in mammals birds, and fish. Receptors which can be considered to be clear orthologs to specific receptors of the DECTIN-1 cluster seem to be limited to mammals. However, within the class of mammals, most C-type lectin-like receptors of the DECTIN-1 cluster seem to be highly conserved in both structure and function.

##  Conflict of Interests

H. Ghadially is currently employed by MedImmune Ltd, Cambridge, UK. However, MedImmune had no role in study design, data collection and analysis, decision to publish, or preparation of the paper.

## Figures and Tables

**Table 1:** Members of the DECTIN-1 cluster in different mammalian families.

	Primate	Bovine	Porcine	Murine	Canine	Marsupial	Monotreme
DECTIN-1	*✓✓*	*✓*	*✓*	*✓✓*	~	?	?
LOX-1	*✓✓*	*✓✓*	*✓*	*✓✓*	~	?	~
CLEC-1	*✓*	~	?	*✓✓*	~	~	?
CLEC-2	*✓✓*	~	?	*✓✓*	~	~	?
CLEC9A	*✓✓*	~	?	*✓*	~	?	?
CLEC12B	*✓✓*	~	?	*✓✓*	~	?	~

Existence and available information. *✓✓*: identified and function characterized, *✓*:  identified and some information published, ~: gene/protein predicted only, ?: unknown.

**Table 2:** Ligands, functions and protein sequence conservation of members of the DECTIN-1 cluster.

	Ligand	Function	% Sequence identity to human protein					
			Chimp	Cow	Mouse	Dog	Platypus	Opossum
DECTIN-1	*β*-glucan	Phagocytosis, cytokine production, antifungal immune defense	98	74	61	74	—	—

LOX-1	Modified lipoproteins, apoptotic cells, bacteria	Endocytosis, phagocytosis, apoptosis, cytokine production, ROS production, implications in vascular disease	99	73	61	74	35	—

CLEC-1	Unknown	Putative regulatory function	99	76	70	75	—	74 (CTLD only)

CLEC-2	Rhodocytin, podoplanin	Platelet activation, implications in tumor metastasis, lymphatic/blood vessel separation during development, phagocytosis, cytokine production	97	69	65	70	—	74 (CTLD only)

CLEC9A	Unknown ligand on necrotic cells	Cross-presentation of dead-cell-associated antigens to T cells	99	71	60	72	—	—

CLEC12B	Unknown	Putative inhibitory function	?	80	72	81	26	—

Sequence identity to the human ortholog was obtained using the NCBI HomoloGene tool (available at: http://www.ncbi.nlm.nih.gov/sites/entrez?db=homologene).
